# Dl-3-n-Butylphthalide Ameliorates Diabetic Nephropathy by Ameliorating Excessive Fibrosis and Podocyte Apoptosis

**DOI:** 10.3389/fphar.2021.628950

**Published:** 2021-08-23

**Authors:** Jingyu Xu, Zonghao Tang, Youwu He, Shufang Cai, Beini Wang, Susu Zhang, Man Wu, Kai Qian, Kailun Zhang, Bo Chai, Guorong Chen, Ke Xu, Hao Ji, Jian Xiao, Yanqing Wu

**Affiliations:** ^1^ The Institute of Life Sciences, Wenzhou University, Wenzhou, China; ^2^ Drug Discovery Research Center, Key Laboratory of Medical Electrophysiology of Ministry of Education, Southwest Medical University, Luzhou, China; ^3^ Department of hand and plastic surgery, The First People’s Hospital of Yuhang District, Hangzhou, China; ^4^ Molecular Pharmacology Research Center, School of Pharmaceutical Science, Wenzhou Medical University, Wenzhou, China; ^5^ Department of Pathology, The First Affiliated Hospital of Wenzhou Medical University, Wenzhou, China

**Keywords:** diabetic nephropathy, dl-3-n-butylphthalide, podocytes, endoplasmic reticulum stress, glomerular filtration barrier, vascular endothelial growth factor

## Abstract

Diabetic nephropathy (DN) is a common diabetes associated complication. Thus, it is important to understand the pathological mechanism of DN and find the appropriate therapeutic strategy for it. Dl-3-n-Butylphthalide (DL-NBP) has anti-inflammatory and antioxidant effects, and been widely used for the treatment of stroke and cardiovascular diseases. In this study, we selected three different doses (20, 60, and 120 mg⋅kg^−1^ d^−1^) of DL-NBP and attempted to elucidate its role and molecular mechanism underlying DN. We found that DL-NBP, especially at the dose of 60 or 120 mg⋅kg^−1^ d^−1^, could significantly ameliorate diabetes-induced elevated blood urea nitrogen (BUN) and creatinine level, and alleviate renal fibrosis. Additionally, the elevated expressions of collagen and α-smooth muscle actin (*α*-SMA) in the kidney from db/db mice were found to be significantly suppressed after DL-NBP treatment. Furthermore, mechanistic studies revealed that DL-NBP inhibits pro-inflammatory cytokine levels, thereby ameliorating the development of renal fibrosis. Moreover, we found that DL-NBP could not only reduce the endoplasmic reticulum stress (ERS), but also suppress activation of the renin-angiotensin system to inhibit vascular endothelial growth factor (VEGF) level, which subsequently reduces the podocyte apoptosis in kidney of db/db mice. In a word, our findings suggest that DL-NBP may be a potential therapeutic drug in the treatment of DN.

## Introduction

Diabetic nephropathy (DN) is a microvascular complication of diabetes that can lead to end-stage renal disease (ESRD). Globally, about 40% of patients with diabetes will develop into DN; therefore, it is an urgent need to find the appropriate and effective treatment for DN (Hu et al., 2015). The main features of DN include thickening of the glomerular basement membrane, mesangial dilatation, damage to the podocytes and tubular cells, sclerosis, and renal tubular interstitial fibrosis (Fan et al., 2017). There are multi-factors involved in the development of DN. Hyperglycemia, inflammation, endoplasmic reticulum stress (ERS), and disorder of renin-angiotensin system (RAS) are the important causative factors for the occurrence and development of DN (Ruggenenti et al., 2010; Kanwar et al., 2011; Navarro-González et al., 2011).

As it is known, RAS plays a key role in the development of DN, which can increase glomerular capillary pressure and permeability, stimulate the proliferation and hypertrophy of renal cells, and affect the function of glomerular filtration barrier (GFB) (Guo et al., 2017). Previous studies suggest that RAS inhibitors, including angiotensin-converting enzyme inhibitors and angiotensin receptor blockers, can effectively inhibit the progression of renal disease (Alfie et al., 2011). Podocytes, the epithelial cells of the renal sacs, attach to the lateral surface of the glomerular basement membrane and form the GFB along with the vascular endothelial cells. Podocytes are involved in maintaining the function of GFB, and podocyte damage leads to proteinuria (Shih et al., 1999). Activated RAS promotes angiotensin Ⅱ (Ang-II) expression in individuals with diabetes (Ribeiro-Oliveira et al., 2008). During diabetic condition, elevated Ang Ⅱ has been reported to induce the vascular endothelial growth factor (VEGF) overexpression, podocyte damage, and GFB destruction (Mironidou-Tzouveleki et al., 2011; Zheng et al., 2019).

ER is the primary organelle involved in the synthesis, folding, and modification of proteins. ERS is caused by a disorder in protein folding under pathological conditions and subsequently activates an adaptive reaction, unfolded protein response (UPR) (Inagi et al., 2014). Excessive and prolonged activation of UPR may lead to cell damage and death (Cunard and Sharma, 2011). Excessive ERS and inflammation have also been found to be associated with podocyte damage (Watanabe et al., 2014; Fan et al., 2017).

Butylphthalide (NBP), a compound derived from celery, has been approved by the China Food and Drug Administration for treating acute ischemic strokes. NBP is a chiral molecule having the stereoisomers L, DL, and D-NBP (Li et al., 2010). DL-NBP has the feature with multi-target complexity and exerts several pharmacological effects, such as microcirculation reconstruction, anti-inflammation, and ameliorating cellular stress (Ye et al., 2015; Liao et al., 2018; Luo et al., 2019). DL-NBP has been widely used for the treatment of nervous system disease (Chen et al., 2019). Recently, it is also found that DL-NBP can effectively delay the progression of hypertensive nephropathy, which can reduce tubular damage, excessive inflammation and oxidative stress in hypertensive condition (Zhu et al., 2015). In diabetic condition, the body is exposed to excessive oxidative stress (Kahleova et al., 2011), inflammation (Liang et al., 2018), elevated ERS (Xu et al., 2017), and other adverse conditions. These findings suggest that DL-NBP can be considered as a potential drug for the treatment of DN. We hypothesized that DL-NBP treatment could ameliorate the development of DN. In our current study, we used a db/db mice model of type 2 diabetes to determine the role and molecular mechanism of DL-NBP in DN.

## Materials and Methods

### Animal Experiments

**FIGURE 1:**
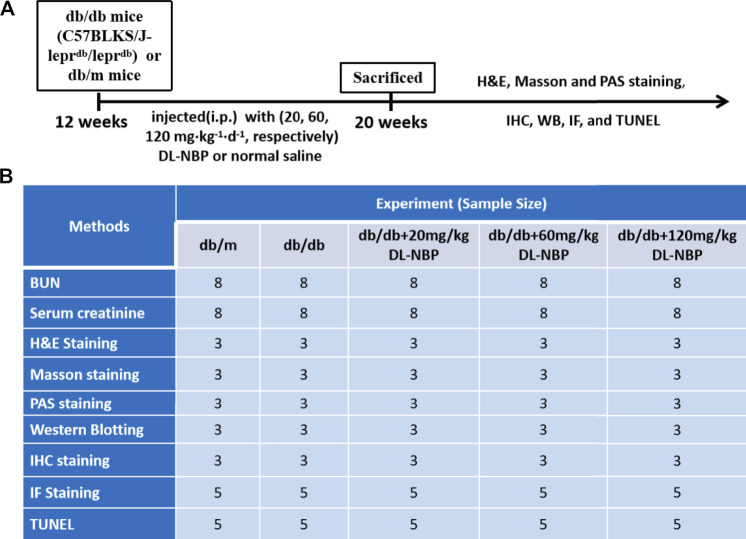
Experimental protocol of DL-NBP treating for DN. **(A)** Duration of the animal study; **(B)** A table containing the group of each experiment and its sample size. BUN, blood urea nitrogen; H&E, hematoxylin and eosin; TUNEL, TdT-mediated dUTP Nick-End Labeling; WB, Western blotting; IF, immunofluorescence staining; IHC, Immunohistochemistry staining

Twelve-week-old male db/db mice (C57BLKS/J-lepr^db^/lepr^db^) and their nondiabetic db/m littermates were purchased from the Model Animal Research Center of Nanjing University (Nanjing, China). All experimental procedures were approved by the Institutional Animal Care and Use Committee of Wenzhou Medical University according strictly to the National Institutes of Health Guide. The animals were housed in a room maintained at 22 ± 2.0°C and 50 ± 5% humidity and subjected to a 12-h light/12-h dark cycle before the study. All mice were given access to food and water *ad libitum*. The experimental design is presented in Figures 1A,B. The db/db mice were randomly divided into four groups, of which db/db mice in three groups were intraperitoneally (i.p.) injected with 20, 60, 120 mg⋅kg^−1^ d^−1^ of DL-NBP, respectively, for 8 weeks. The rest of the db/db mice and db/m mice were injected daily with the same volume of saline. After 8 weeks, the mice were anesthetized with isoflurane at a concentration of 1.5%. The serum and kidneys were collected for biochemical and molecular analysis. The serum was used to detect the level of blood urea nitrogen (BUN, C013-2-1) and creatinine (Cr, C011-1-1) using the respective assay kits from Nanjing Jiancheng Bioengineering Institute.

### Hematoxylin and Eosin (H&E) Staining

The kidneys were collected and fixed with 4% paraformaldehyde (PFA) in phosphate-buffered saline (PBS), then dehydrated using alcohol and immersed in xylene. Next, the sample tissues were embedded in paraffin and cut into 5 μm thick section. These kidney sections were stained with H&E reagent following the manufacturer’s instructions (Beyotime, C0105) and observed using a Carl Zeiss microscope.

### Masson’s Trichrome Staining and Periodic Acid-Schiff Staining

Masson’s trichrome staining and PAS staining were used to study the deposition of collagen and glycogen in kidney. After dewaxing and rehydration, kidney sections (5-μm thickness) were prepared and stained with Masson’s trichrome staining kits according to the manufacturer’s instructions (Solarbio, G1345). The images were captured using a Carl Zeiss microscope. For PAS staining, the sections of kidney were dewaxed in water and stained with periodic acid-Schiff (PAS) stain (Solarbio, G1281). After sealing, glycogen accumulation (red positive point) in the kidney was observed using a Leica Microscope (Germany).

### TdT-Mediated dUTP Nick-End Labeling Staining

The ApopTag Fluorescein Direct *In Situ* Apoptosis Detection Kit (Beyotime, C1086) was used to the detect the apoptosis level. The frozen sections were fixed with precooled acetone for 15 min and washed with PBS for three times. The sections were incubated with 20 μg/ml proteinase K working solution for 15 min at 37°C. The slides were then rinsed with PBS for three times and incubated with TUNEL reaction mixture for 1 h at 37°C. The slides were again rinsed with PBS three times and then stained with 4′, 6-diamidino-2-phenylindole (DAPI, Beyotime, Shanghai, China) for 5 min at 25°C. Lastly, the slides were mounted with an aqueous mounting medium and observed using a Leica Microscope (Germany) and imaged.

### Immunohistochemistry and Immunofluorescence Analysis

Briefly, sections (5-μm thickness) were deparaffinized, rehydrated, and incubated in 3% H_2_O_2_ for 15 min. Next, they were subjected to microwave for antigen retrieval in 10 mM sodium citrate and then incubated in blocking solution (5% BSA) for 30 min. For immunohistochemistry, the sections were incubated at 4°C overnight with the following primary antibodies: *α*-SMA (1:500, Abcam, ab32575), Collagen Ⅰ (1:200, Zen bio, 343,277), Collagen Ⅲ (1:200, Zen bio, 250,064), CHOP (1:200, Proteintech, 15204-1-AP), and VEGF (1:200, Proteintech, 15204-1-AP). After washing with PBS with Tween-20 detergent (PBST), the sections were incubated with horseradish peroxidase-labeled secondary antibodies (1:200; Beyotime, A0216 or A0208) for 2 h at 37°C. Lastly, the sections were reacted with 3, 3′-diaminobenzidine (DAB) and observed under a Carl Zeiss microscope. For immunofluorescence analysis, the sections were incubated at 4°C overnight with the following primary antibodies: Nephrin (Affinity, AF7951), TNF-α (1:200, Abcam, ab1793), Cleaved Caspase-3 (1:200, Cell Signaling Technology. 9,664). Then, the sections were incubated with Alexa Fluor 594 AffiniPure goat anti-mouse or Alexa Fluor 488 AffiniPure goat anti-rabbit secondary antibody for 4 h. Subsequently, the cell nuclei were stained with DAPI. Then, the sections were sealed and observed using a Carl Zeiss microscope.

### Western Blotting

Renal tissues were lysed in cell lysates containing the protease inhibitor cocktail (Beyotime, P1005). Equal amounts of the prepared protein were resolved using SDS-PAGE and transferred onto PVDF membranes. After blocking with 5% milk for 1 h, the membranes were blocked with the following primary antibody diluted 1:1,000 (prepared with PBS) at 4°C overnight: Collagen I (Zen-bio, 343,277), Collagen Ⅲ (Zen-bio, 250,064), p-PERK (Affinity, DF7576), p-IRE1α (Affinity, DF8322), GRP78 (Proteintech, 11587-1-AP), ATF-6 (Proteintech, 24169-1-AP), CHOP (Cell Signaling Technology, 2895S), TNF-α (Proteintech, 17590-1-AP), IL-6 (Santa Cruz Biotechnology, sc-57315), IL-10 (Proteintech, 20850-1-AP), VEGF (Proteintech, 15204-1-AP), ACE (Proteintech, 21115-1-AP), AT1 (Abcam, EPR3873), and β-actin (Solarbio, K200058M). The membranes were washed with PBST for three times and then treated with horseradish peroxidase-conjugated secondary antibodies (1:2000; Beyotime, A0216 or A0208) for 2 h at room temperature. Signals were visualized using the ChemiDocXRS + Imaging System (Bio-Rad). All experiments were performed in triplicate and tissue samples were prepared independently for each experiment. The densitometric values of the bands were obtained using Image J software (NIH, Bethesda, MD) and subjected to statistical analysis.

### Statistical Analyses

Data were presented as mean ± SEM. Experiments were performed at least three times and the renal tissues from each replicate were from different mice. GraphPad Prism five was used to determine statistical differences using one-way analysis of variance (ANOVA) followed by the Tukey test. Image J software was used to quantify the expression of protein during Immunohistochemistry and immunofluorescence analysis. Statistical significance was accepted when *p* < 0.05.

## Results

### DL-3-N-Butylphthalide Treatment Mitigates Diabetes-Induced Renal Injury

**FIGURE 2:**
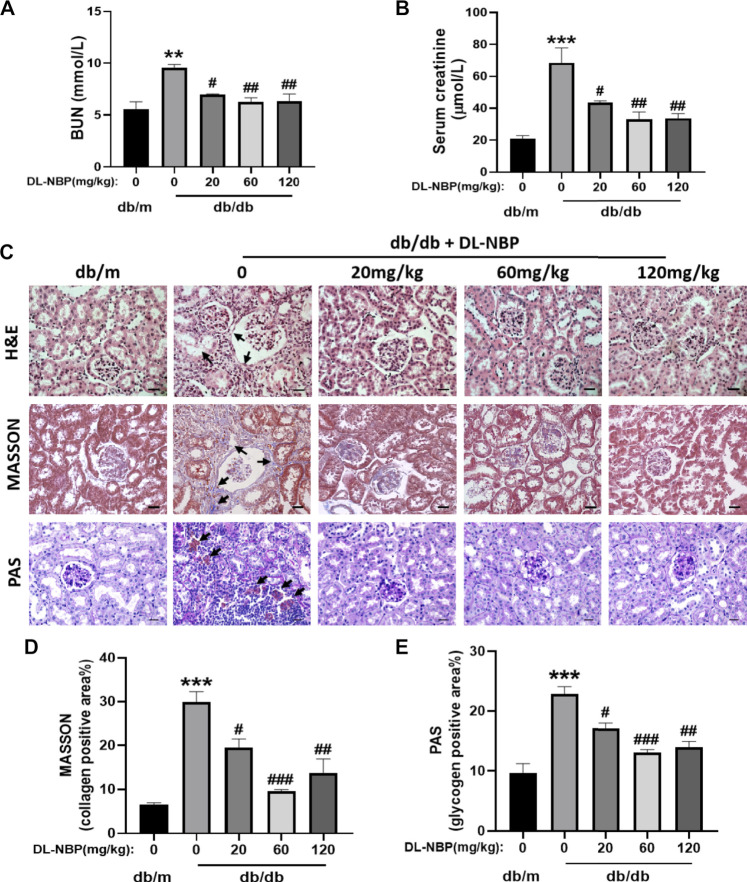
DL-NBP treatment ameliorates renal injury in db/db mice. **(A,B)** BUN and Cr levels in serum from db/m mice, db/db mice, and DL-NBP treated mice (db/db mice were treated with 20, 60, or 120 mg⋅kg^−1^ d^−1^ of DL-NBP) (*n* = 8). **(C–E)** Representative images and quantification of H&E, Masson’s trichrome, and PAS staining (original magnification × 400) in kidney of mice from each group (*n* = 3), Scale bar = 20 μm. Data are presented as the mean ± SEM; **p < 0.01, ***p < 0.001 vs. db/m control mice; ^#^ *p* < 0.05, ^##^ *p* < 0.01, ^###^ *p* < 0.001 vs. db/db mice. BUN, blood urea nitrogen; Cr, creatinine; H&E, hematoxylin and eosin.

After treating with DL-NBP for 8 weeks, we measured the level of BUN and Cr in serum. It was worth noting that the average level of BUN and Cr in serum from db/db mice were significantly higher than those in db/m mice (Figures 2A,B), which suggested that chronic hyperglycemia is likely to cause the dysfunction of kidney. Interestingly, these adverse effects of diabetes were significantly attenuated by DL-NBP treatment (Figures 2A,B). During morphological analysis, we found the serious inflammatory cell infiltration and capillary collapse in the kidney from db/db mice (Figure 2C). Additionally, Masson’s staining (blue) revealed a significant increase of renal interstitial fibrosis in db/db mice (Figures 2C,D). Meanwhile, PAS staining (red) revealed the obvious glycogen deposition around the renal interstitium in db/db mice (Figures 2C,E). More importantly, DL-NBP treatment attenuated these effects of diabetes on kidney (Figures 2C–E).

### DL-3-N-Butylphthalide Treatment Ameliorates Diabetes-Induced Renal Fibrosis

**FIGURE 3:**
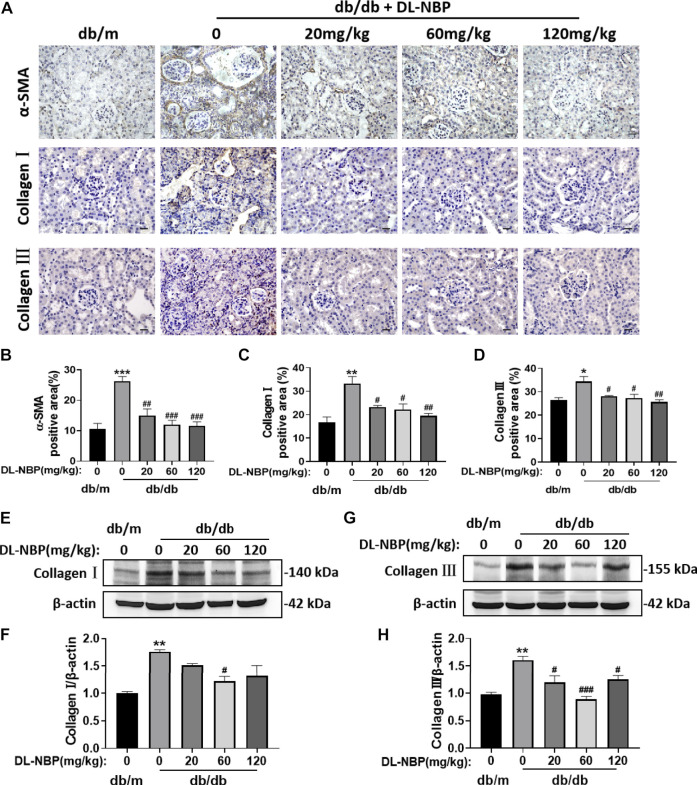
DL-NBP treatment ameliorates diabetes-induced renal fibrosis. **(A–D)** Immunohistochemical staining (original magnification × 400) and quantification of α-SMA, collagen I, and collagen III in kidney of mice from each group (*n* = 3). Scale bar = 20 μm. **(E–H)** Western blotting and quantification of collagen I and collagen III from the renal tissue of each group (*n* = 3). Data are presented as the mean ± SEM. *p < 0.05, **p < 0.01, ***p < 0.001 vs. db/m control group; ^#^ *p* < 0.05, ^##^ *p* < 0.01, ^###^ *p* < 0.001 vs. db/db mice. α-SMA, α-smooth muscle actin.

During the development of DN, type I and III collagen is shown abnormal accumulation and deposition in the extracellular matrix, which subsequently results in renal interstitial fibrosis. The immunohistochemistry results from our study indicated significantly elevated α-SMA, Collagen I and Collagen Ⅲ accumulation around the renal interstitium in db/db mice and these phenomena were alleviated by DL-NBP treatment (Figures 3A–D). Consistent with immunohistochemistry results, the western blotting result further suggested that DL-NBP treatment inhibits diabetes-induced over-expression of type I and III collagen (Figures 3E–H). The above results indicated that DL-NBP ameliorates renal interstitial fibrosis in db/db mice.

### DL-3-N-Butylphthalide Prevents Diabetes-Induced Excessive Inflammation in Kidney

**FIGURE 4:**
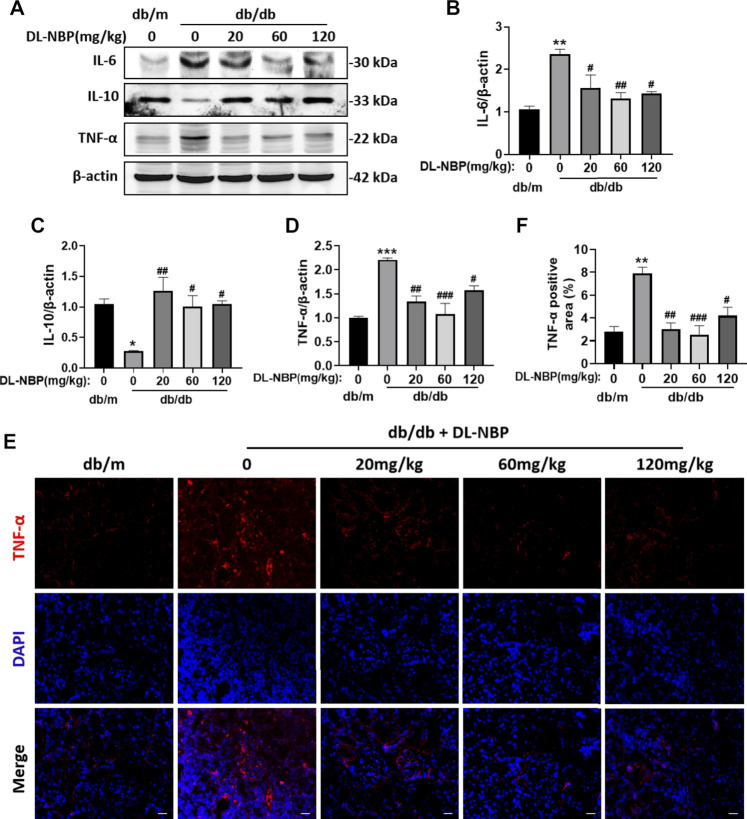
DL-NBP prevents diabetes-induced excessive inflammation in kidney*.* **(A–D)** Western blotting and quantification of IL-6, IL-10 and TNF-α in kidney of mice from each group (*n* = 3). Protein expression was normalized to the expression of β-actin. **(E,F)** The immunofluorescence staining and quantification (original magnification × 400) of TNF-α in kidney of mice from each group (*n* = 3). Scale bar = 20 μm. All data are presented as the mean ± SEM. *p < 0.05, **p < 0.01, ***p < 0.001 vs. db/m control; ^#^ *p* < 0.05, ^##^ *p* < 0.01, ^###^ *p* < 0.001 vs. db/db mice.

Excessive inflammation is recognized as an important factor during the development of DN. We observed that db/db mice shows obvious and excessive inflammation in kidney. Diabetes significantly triggered the increases of pro-inflammatory cytokines (TNF-α and IL-6) and decrease of the anti-inflammatory cytokines (IL-10) (Figures 4A–D). Importantly, the expression of these inflammatory markers in the DL-NBP treated group were consistent with those in the kidney of db/m mice (Figures 4A–D). Furthermore, the result of immunofluorescence staining of TNF-α is consistent with western blotting results (Figures 4E,F). These studies indicated that DL-NBP treatment effectively prevent the excessive inflammation in the kidney of db/db mice.

### DL-3-N-Butylphthalide Treatment Reduces Diabetes-Induced Podocyte Apoptosis in Kidney

**FIGURE 5:**
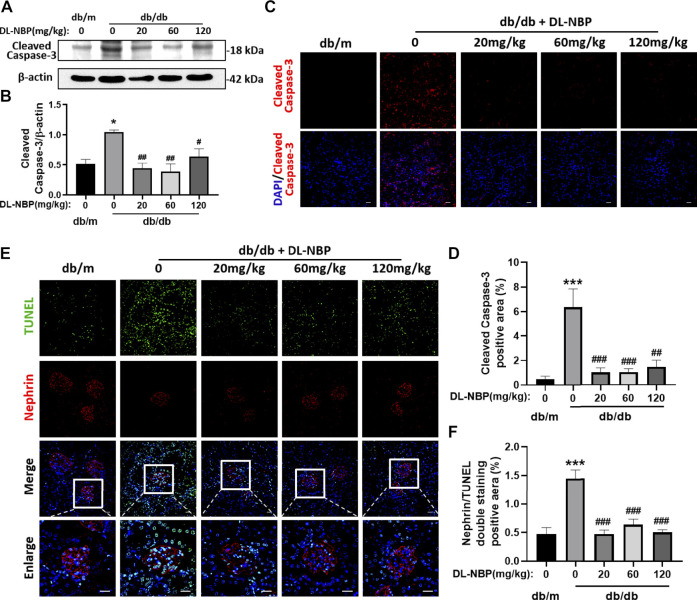
DL-NBP treatment reduces diabetes-induced podocyte apoptosis in kidney. **(A,B)** Western blotting and quantification of Cleaved Caspase-3 expression in each group (*n* = 3). **(C,D)** Immunofluorescence staining of Cleaved Caspase-3 (red) in kidney of db/m (control) mice, db/db mice, DL-NBP mice (db/db mice were treated with 20, 60, or 120 mg⋅kg^−1^ d^−1^ of DL-NBP) (original magnification × 400) (*n* = 5). Scale bar = 20 μm. **(E,F)** Co-staining of nephrin (red) and TUNEL (green) (original magnification × 400) was used to detect the extent of cell apoptosis in each group (*n* = 5). Scale bar = 20 μm. TUNEL, TdT-mediated dUTP Nick-End Labeling. All data are presented as the mean ± SEM. *p < 0.05, ***p < 0.001 vs*.* db/m control; ^#^ *p* < 0.05, ^##^ *p* < 0.01, ^###^ *p* < 0.001 vs. db/db mice.

The excessive apoptosis of podocytes is an important pathological change that occurs during DN. Podocytes play a vital role in maintaining the permeability of GFB. The damage or loss of podocytes leads to proteinuria and glomerular sclerosis (Canaud et al., 2013). We found that Cleaved Caspase-3 (the executive molecule of apoptosis) is significantly enhanced in the kidney from db/db mice and DL-NBP treatment significantly inhibits it (Figures 5A,B). The results of Cleaved Caspase-3 staining is consistent with that in western blotting (Figures 5C,D). In addition, we further performed co-staining of TUNEL staining and nephrin (labeled for podocyte cells) to explore the apoptosis level of podocytes. We found that DL-NBP treatment significantly reduces the number of TUNEL-positive signaling in the podocyte cells of db/db mice (Figures 5E,F). These results suggested that DL-NBP may reduce the loss of podocytes in diabetic mice and protect the GFB.

### DL-3-N-Butylphthalide Treatment Inhibits Diabetes-Induced Elevated Endoplasmic Reticulum Stress in Kidney

**FIGURE 6:**
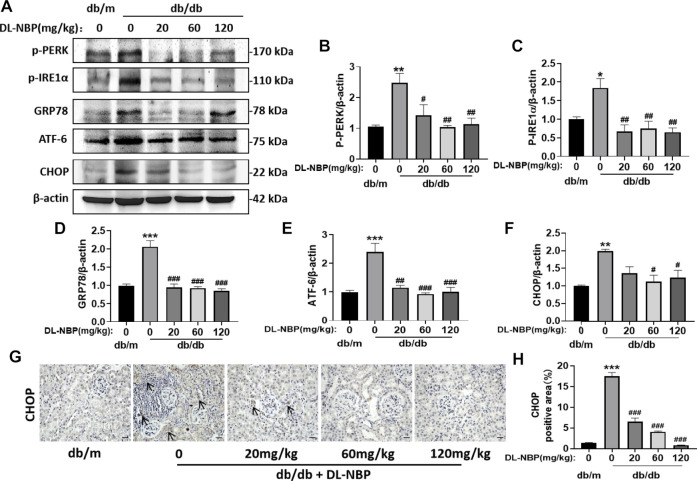
DL-NBP treatment inhibits diabetes-induced elevated ERS in kidney. **(A–F)** Western blotting and quantification of ERS-elevated protein expression [p-PERK **(A,B)**, p-IRE1α **(A,C)**, GRP78 **(A,D)**, ATF-6 **(A,E)**, CHOP **(A,F)**] in kidney of mice from each group (*n* = 3). Protein expression was normalized to the expression of β-actin. **(G,H)** Representative images of immunohistochemical staining of CHOP in kidney of mice from each group (*n* = 3). Scale bar = 20 μm. Data are presented as mean ± SEM; *p < 0.05, **p < 0.01, ***p < 0.001 vs*.* db/m control; ^#^ *p* < 0.05, ^##^ *p* < 0.01, ^###^ *p* < 0.001 vs. db/db mice.

ERS is related to the damage of podocytes, which contributes to damage of glomerular filtration system, resulting in glomerular sclerosis (Daehn et al., 2014; Guo et al., 2017; Tian et al., 2018). CHOP is a key mediator during ERS-induced cell death (Fang et al., 2014; Wang et al., 2015). To determine the role of ERS on DL-NBP for DN treatment, we determined the expression of the ERS-related markers in kidney. Compared with those in db/m mice, the expressions of GRP78, P-PERK, p-IRE1α, ATF-6, and CHOP were significantly increased in the kidney from db/db mice, whereas these increases were reversed by DL-NBP treatment (Figures 6A–F). Furthermore, we found that DL-NBP treatment significantly reduces CHOP-positive signals in the kidney of db/db mice (Figures 6G,H), which is consistent with the result of western blotting. Taken together, our findings suggested that DL-NBP treatment could inhibit ERS in the kidney of db/db mice.

### DL-3-N-Butylphthalide Restores the Function of Renin-Angiotensin System and Blocks Vascular Endothelial Growth Factor Overexpression

**FIGURE 7:**
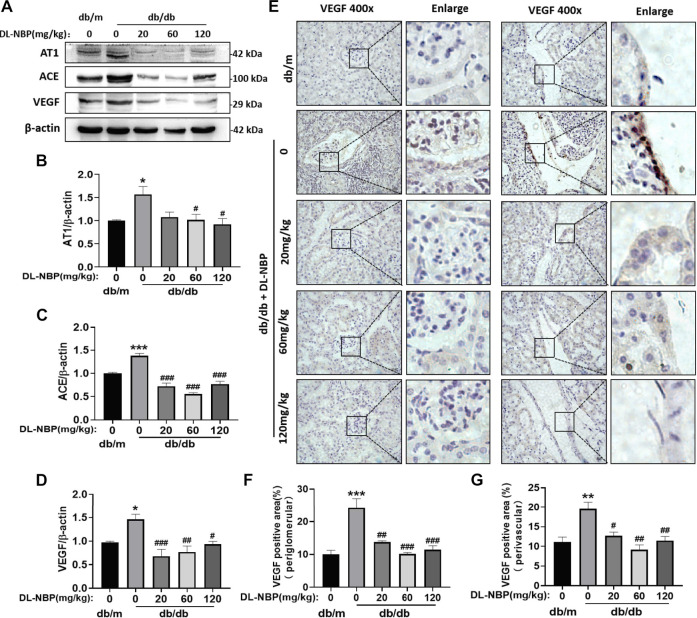
DL-NBP restores the function of RAS and blocks VEGF overexpression. **(A–D)** Western blotting and quantification analysis of AT1, ACE, and VEGF in kidney of mice from each group (*n* = 3). Protein expression was normalized to the expression of β-actin. **(E–G)** Immunohistochemical staining of VEGF (brown positive point) in kidney of mice from each group (original magnification × 400) (*n* = 3). Scale bar = 20 μm. Data are presented as mean ± SEM; *p < 0.05, **p < 0.01, ***p < 0.001 vs. db/m control; ^#^ *p* < 0.05, ^##^ *p* < 0.01, ^###^ *p* < 0.001 vs*.* db/db mice. AT1, angiotensin II-related proteins angiotensin II receptor type 1; ACE, angiotensin II generating enzyme; VEGF, vascular endothelial growth factor.

Diabetes destroys the RAS (Ribeiro-Oliveira et al., 2008). We found that diabetes significantly increases the expressions of angiotensin II receptor type 1 (AT1) and angiotensin II generating enzyme (ACE) in the kidney (Figures 7A–C), which are restored by DL-NBP treatment (Figures 7A–C). It has been reported that the increase in angiotensin II can induce VEGF overexpression (Kang et al., 2006). VEGF is mainly derived from the podocytes and tubular epithelial cells of the kidney, and exerts its function in the paracrine and autocrine mode (Nakagawa et al., 2013). Elevated VEGF causes glomerular basement membrane thickening, podocyte apoptosis, and GFB destruction (Mironidou-Tzouveleki et al., 2011). We found that the VEGF level in the kidney of db/db mice is higher than that in the other groups (Figures 7A,D). Furthermore, VEGF staining showed that its expression is significantly increased in the lateral podocytes of the glomerular basement membrane and vascular endothelial cells in the db/db group when compared to that in db/m mice (Figures 7E–G). Importantly, DL-NBP treatment was found to ameliorate this increase of VEGF in the kidney from db/db mice (Figures 7E–G), suggesting that the efficacy of DL-NBP in DN may be attributed to restore the function of RAS and consequently normalize VEGF expression in kidney.

## Discussion

We used the db/db mice as a type 2 diabetes model and selected three different doses of DL-NBP (20, 60, and 120 mg⋅kg^−1^ d^−1^) to treat db/db mice for 8 weeks. We found that diabetic mice exhibits renal dysfunction with manifesting an increase in the levels of BUN and Cr. Interestingly, these effects were significantly reversed after the administration with DL-NBP. No significant differences (*p* < 0.01) in efficacy were found between treatment with 60 and 120 mg⋅kg^−1^ d^−1^ of DL-NBP; however, both these doses of DL-NBP were more efficacious than the 20 mg⋅kg^−1^ d^−1^ of DL-NBP(*p* < 0.05, Figures 2A,B). Furthermore, we found that DL-NBP treatment significantly ameliorates renal tubulointerstitial fibrosis and the excessive apoptosis of podocytes in db/db mice (Figures 2C–E, and Figure 5). These results strengthened our hypothesis that DL-NBP exerts a protective role in DN.

Severe cell damage resulted from inflammation, hypoxia, and oxidative stress in diabetes may lead to ER stress, and lastly lead to the apoptosis of podocytes in kidney (Inagi, 2009). A study has reported that tauroursodeoxycholic acid treatment suppresses ERS and reduces its damage for podocytes in diabetic mice (Fan et al., 2017). Emodin treatment also reverses ERS-mediated podocyte apoptosis by inhibiting the PERK/eIF2α axis in DN (Tian et al., 2018). These results suggest that ERS may be a potential target for DL-NBP in the treatment of DN. In our current study, we found that DL-NBP significantly suppresses the expressions of the ERS-related makers (p-PERK, ATF-6, GRP78, p-IRE1α and CHOP). More importantly, treatment with 60 and 120 mg⋅kg^−1^ d^−1^ of DL-NBP have a better effect during ameliorating ERS than that in 20 mg⋅kg^−1^ d^−1^ of DL-NBP (Figure 6). Our current results suggest that DL-NBP may protect podocytes against injury by inhibiting elevated ERS during DN.

Inflammation is considered as a central factor in the development of DN (Liang et al., 2018). Several components in the diabetic condition, such as hyperglycemia, RAS disorder, and oxidative stress, can activate the inflammatory processes in kidney (Duran-Salgado and Rubio-Guerra, 2014). This effect subsequently causes monocytes and lymphocytes to infiltrate organs, and secretes harmful substances (Duran-Salgado and Rubio-Guerra, 2014). In our current study, the db/db mice showed severe inflammatory response with an increases of the pro-inflammatory cytokines (TNF-α and IL-6), which were significantly ameliorated after DL-NBP treatment (Figure 4). It is worth mentioning that 60 mg⋅kg^−1^ d^−1^ of DL-NBP exerts a better anti-inflammatory effect on the DN treatment (Figure 4). Collectively, these results suggested that an increase of anti-inflammatory function may also be a key factor in DL-NBP treatment for DN.

VEGF (especially, the specific subtype VEGF-A165A), an effective vascular activator, can increase vasodilation and vascular permeability and promote angiogenesis (Bates, 2010). It has been reported that DL-NBP treatment promotes the functional recovery after a focal transient ischemic stroke by enhancing VEGF expression and increasing the number of microvessels (Wang et al., 2019). Moreover, DL-NBP also inhibits excessive apoptosis and plays a protective role in diabetic brain injury by increasing VEGF expression (Zhang et al., 2010). However, excessive VEGF may also lead to adverse functional consequences of vascular lesions in individuals with diabetes. It has been reported that VEGF is increased in the kidney during DN development (Zheng et al., 2019). Blocking the VEGF signaling pathway reduces the proteinuria in diabetic mice (Chen and Ziyadeh, 2008). Another study has reported that treatment with anti-VEGF protein in the early stages of diabetes can ameliorate renal dysfunction (Yang et al., 2003). Consistent with prior study, our findings showed that diabetes triggers the increase of VEGF in kidney, and that DL-NBP treatment could normalize this increase (Figures 7A, D–G).

RAS is involved in the regulation of blood pressure, fluid and electrolyte balance, and systemic vascular resistance. RAS induced-elevated Ang-II and VEGF together play an important role in the pathogenesis of DN (Kang et al., 2006). The STAT3 inhibitor (S3I-201) ameliorates RAS activation and reduces the local production of VEGF in podocytes and renal tubule epithelium, which are induced by elevated ACE levels in diabetes (Zheng et al., 2019). In addition, Ang-II levels in the proximal renal tubular epithelial cells are increased in mice during hyperglycemia, resulting in elevated reactive oxygen species (ROS) and activation of the ERK pathway by inducing VEGF expression (Feliers et al., 2006; Feliers and Kasinath, 2010). In our current study, we also found that the incidence of diabetes significantly triggers the increases of ACE and AT1, indicating an increase in the generation of Ang-II. Consistent with the findings of previous study, our study showed that the VEGF level in kidney is remarkably increased in db/db mice. Interestingly, DL-NBP could significantly suppress the expression of AT1, ACE, and VEGF in kidney of db/db mice (Figure 7).

VEGF is mainly produced by podocytes in kidney (Canaud et al., 2013). In diabetic individuals, VEGF can stimulate the production of collagen, cause the glomerular base to thicken, and inhibit the expression of nephrin, consequently leading to the loss of podocytes (Wolf et al., 2005). Podocytosis may reflect the downregulation of VEGF in these stages of renal disease (Bortoloso et al., 2001). This reduction in VEGF is likely relative, rather than absolute, because living podocytes can promote the overexpression of VEGF in a diabetic condition (Mironidou-Tzouveleki et al., 2011). We found that treatment with DL-NBP could significantly reduce the expression of VEGF. DL-NBP itself may not have an anti-VEGF effect, but it can inhibit ERS and inflammation, reduce the activation of RAS and eventually suppress VEGF, and subsequently protect the normal function of podocytes.

Inflammation, ERS and RAS system disorder are the main molecular mechanisms during DN development. During DN development, there are cross-talking among these mechanisms. It is reported that the expression and activity of Renin (expressed and secreted by juxtaglomerular apparatus (JGA) cells) are regulated by certain inflammatory factors (such as IL-6, IL-1β, and TNF-α, etc.) (Todorov et al., 2004; Pan et al., 2005; Liu et al., 2006). The activation of Ang-Ⅱ is involved in the inflammatory response and tissue remodeling of DN (Mezzano et al., 2003). Additionally, it is been pointed out that the ERS inhibitor (4-PBA) can inhibit the inflammatory cascade and prevent the progression of DN (Qi et al., 2011). Therefore, there may also be the mutual regulationship among these mechanisms during treatment of DN with DL-NBP, which need to be explored in the following study.

**FIGURE 8:**
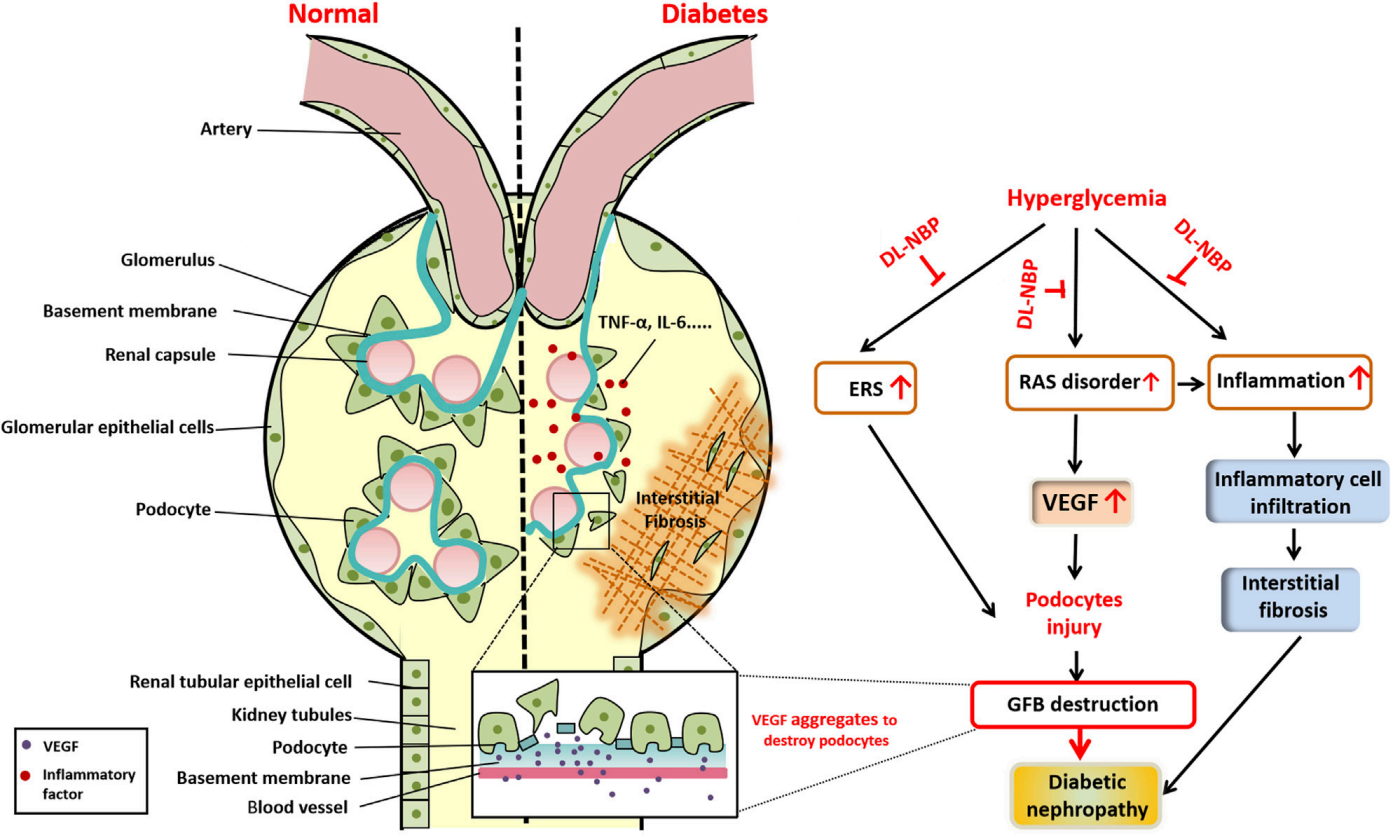
Schematic showing the role and mechanism of DL-NBP in DN. Diabetes significantly induces renal interstitial fibrosis and podocyte apoptosis. The mechanistic study reveals that diabetes triggers ERS, induces excessive inflammation, and leads to RAS disorder, ultimately resulting in podocyte apoptosis. DL-NBP treatment significantly reverses these effects and ameliorates DN. DN, diabetic nephropathy; ERS, endoplasmic reticulum stress; GFB, glomerular filtration barrier; RAS, renin-angiotensin system; VEGF, vascular endothelial growth factor.

To summarize, we used a db/db mice model to determine the efficacy of DL-NBP in DN treatment. Three different doses of DL-NBP (20, 60, and 120 mg⋅kg^−1^ d^−1^) were chosen to treat db/db mice. We found that DL-NBP, especially at a dose of 60 mg⋅kg^−1^ d^−1^, has a better therapeutic effect in DN treatment. It significantly decreased the BUN and Cr levels and ameliorated the extent of fibrosis in db/db mice. Mechanistic studies further revealed that inflammation, ERS, and RAS disorders are the main regulatory mechanisms during the development of DN (Figure 8). DL-NBP treatment significantly reversed renal interstitial fibrosis and the excessive podocyte apoptosis in kidney of db/db mice by inhibiting the elevated inflammation, ERS and RAS disorder.

## Data Availability

The raw data supporting the conclusion of this article will be made available by the authors, without undue reservation.

## Abbreviations

DN, diabetic nephropathy; DL-NBP, dl-3-n-Butylphthalide; BUN, blood urea nitrogen; Cr, creatinine; TUNEL, TdT-mediated dUTP Nick-End Labeling; IF, immunofluorescence staining; IHC, Immunohistochemistry staining; α‐SMA, α-smooth muscle actin; ERS, endoplasmic reticulum stress; RAS, renin-angiotensin system; VEGF, vascular endothelial growth factor; ESRD, end-stage renal disease; GFB, glomerular filtration barrier; NBP, butylphthalide; PFA, paraformaldehyde; PBS, phosphate buffered saline; H&E, hematoxylin and eosin; AT1, angiotensin II-related proteins angiotensin II receptor type 1; ACE, angiotensin II generating enzyme; UPR, unfolded protein response; TUDCA, tauroursodeoxycholic acid; Ang-II, angiotensin II; ROS, reactive oxygen species; IL-6, interleukin-6; IL-10, interleukin-10; TNF-α, tumor necrosis factor-α.
